# iPSC-cardiomyocytes in the preclinical prediction of candidate pharmaceutical toxicity

**DOI:** 10.3389/fphar.2024.1308217

**Published:** 2024-02-28

**Authors:** Tim Y. T. Lee, John G. Coles, Jason T. Maynes

**Affiliations:** ^1^ Program in Molecular Medicine, SickKids Research Institute, Toronto, ON, Canada; ^2^ Department of Biochemistry, University of Toronto, Toronto, ON, Canada; ^3^ Program in Translational Medicine, SickKids Research Institute, Toronto, ON, Canada; ^4^ Department of Cardiovascular Surgery, The Hospital for Sick Children, Toronto, ON, Canada; ^5^ Department of Anesthesia and Pain Medicine, Hospital for Sick Children, Toronto, ON, Canada; ^6^ Department of Anesthesiology and Pain Medicine, University of Toronto, Toronto, ON, Canada

**Keywords:** ipsc-cm, cardiotoxicity, cardiac, preclinical, pharmaceutical, drug discovery

## Abstract

Many challenges remain in the preclinical evaluation, adjudication, and prioritization of novel compounds in therapeutic discovery pipelines. These obstacles are evident by the large number of candidate or lead compounds failing to reach clinical trials, significantly due to a lack of efficacy in the disease paradigm of interest and/or the presence of innate chemical toxicity. The consequential compound attrition in discovery pipelines results in added monetary and time costs, potential danger to patients, and a slowed discovery of true therapeutics. The low rate of successful translation calls for improved models that can recapitulate *in vivo* function in preclinical testing to ensure the removal of toxic compounds earlier in the discovery process, in particular for the assessment of cardiotoxicity, the leading cause of post-market drug withdrawal. With recent advances in the development of human Inducible pluripotent stem cell derived cardiomyocytes (iPSC-CMs), novel compounds can be assessed with better disease relevance while more accurately assessing human safety. In this review, we discuss the utility of iPSC-CMs in preclinical testing by taking advantage of the inherent ability to mimic CMs *in vivo*. We explore the similarities and differences in electrophysiology, calcium handling, cellular signaling, contractile machinery, and metabolism between iPSC-CMs and adult CMs as these complex coordinated functions directly relate to toxicity evaluation. We will highlight considerations when using iPSC-CMs, such as maturation protocols, to ensure a more representative phenotype of the adult human CM, and how different populations of CMs can affect results in compound testing.

## 1 Introduction

The predictive and accurate preclinical evaluation of novel compounds in therapeutic discovery pipelines remains a considerable challenge, illustrated by the high rate of lead compounds failing to reach clinical implementation. A consequential contributor to this high failure rate is the lack of proper assessment of toxicity and off-target effects in highly translational and physiologically relevant pre-clinical models. The resulting attrition of compounds incurs significant monetary and time costs but also poses potential risks to those in need of the therapeutic (Seyhan, 2019). Evidence of post-market withdrawal due to cardiotoxicity has been reported in several studies. In an analysis of 121 withdrawn medicinal products between 1960–1999, 8.7% of the withdraws were due to unanticipated cardiotoxicity (Fung et al., 2001). Another study identified that 63 out of 462 (14%) of withdrawn medicinal products from 1953–2013 were due to unexpected adverse cardiovascular effects (Onakpoya et al., 2016). More recently, an analysis of 78 drugs with known side effects on cardiovascular function yielded that 27 of these compounds were withdrawn from the market due to overt cardiotoxicity (Mamoshina et al., 2021). Examples of post-market withdrawal for cardiotoxicity include dronedarone, an effective therapeutic for treating atrial fibrillation that had proved its disease efficacy in a double blinded, multi-center, randomized trial (Singh et al., 2007), but was later found to increase the mortality in patients predominantly due to the exacerbation of pre-existing heart failure (Køber et al., 2008). More commonly known and notorious cardiotoxic drugs include the anthracycline class of anticancer pharmaceuticals, which are still utilized in oncology (Di Marco et al., 1964) despite known drug-induced cardiac dysfunction (Tan et al., 1967). Unknown and unanticipated cardiotoxic effects extend to numerous therapeutic classes including anti-diabetics (Lee et al., 2022), fluoroquinolone antibiotics (Frothingham, 2001), and selective serotonin reuptake inhibitor antidepressants (Funk and Bostwick, 2013), which all have pro-arrhythmic effects discovered post market release. Pre-clinical models which facilitate evaluation more consistent with *in vivo* human physiology would provide an opportunity to identify compound cardiotoxicity earlier and more accurately in the development pipeline, reducing post-market drug withdrawal and improving patient safety.

The ability to reprogram end-differentiated human cells into pluripotent stem cells with subsequent re-differentiation back into a target tissue provides a platform for assessing novel compounds in a system that more closely resembles the *in vivo* human environment (Takahashi et al., 2007). This technology gave rise to the generation of inducible pluripotent stem cell derived cardiomyocytes (iPSC-CMs) as a model for heart function (Kałużna et al., 2022; Yang et al., 2022). This review explores the similarities and differences between iPSC-CMs and *in vivo* cardiomyocytes (CMs) found in the human heart, and how iPSC-CMs can be used to assess adverse changes to ion channel function, signal transduction, contractile function, and metabolism, as mechanisms for compound toxicity. We review steps to improve model translation, including cell maturation, to more closely mimic the adult human heart. Significantly, clinical cardiotoxicity manifests as changes to heart beating (rate or rhythm) and contractile force (inotropy). While these are inherent phenotypes that can be measured in the *in vivo* heart itself, in isolated cardiomyocytes or cardiac tissue from model species, or in iPSC-CMs, the underlying etiology is primarily related to altered activity (direct or indirect) of ion channels. The delicate and coordinated function of the multitude of ion channels present in the cardiomyocyte requires the presence (expression) and normal activity of each channel, both considerations that are changed naturally during cardiac development and in model systems. Effects on channels affecting the three primary ions (sodium, potassium, and calcium) are important to consider, including how these are represented in the testing system itself, before knowing if they can be evaluated for pharmacologic modification. The calcium cycling machinery is particularly affected in iPSC-CM compared to the *in vivo* environment and significant experimental efforts are made to bring the two closer aligned prior to compound evaluation. Through better cardiotoxicity prediction, iPSC-CMs can provide a highly valuable tool to improve discovery pipeline efficiency and candidate drug safety, advancing therapeutic discovery.

## 2 Functional similarities between iPSC-CMs and the human *in vivo* cardiomyocytes

To enhance the accuracy of predicting potential cardiotoxicity, the electrical and mechanobiology of the iPSC-CMs must mimic the cellular physiology of a CM within the human heart. Although the iPSC-CMs contains a human proteome, developmental stage/maturation-dependent protein expression (i.e., myosin heavy chain isoform) and the general lack of supporting non-cardiomyocyte cell types (i.e., fibroblast, endothelial cell) can affect these metrics and alter responses when testing compound activity.

### 2.1 Electrophysiology and ion channels

iPSC-CMs are electrically active cells, recapitulating the complex ionic current profiles observed in the human endogenous cardiomyocyte that constitute the CM action potential (AP). Characterized currents include depolarizing sodium (*I*_Na_), transient outward potassium (*I*_to_), L-type and T-type calcium (*I*_Ca_), slow and rapid delayed rectifier (*I*_Ks,_ *I*_Kr_), pacemaker (*I*_f_), and the inward rectifying potassium (*I*_K1_) currents. The similarities and differences of these currents between *in vitro* and *in vivo* environments plays an important role in how iPSC-CMs can be used for detecting the potentially arrhythmogenic effects of compounds.

The APs in iPSC-CMs can vary depending on the cell population of a developed beating monolayer, and are classified as either atrial-, nodal-, or ventricular-like cells (Zhang et al., 2009; Zwi et al., 2009; Burridge et al., 2014). The AP differences between the cell constituents of the potentially heterogenous population in iPSC-CMs were delineated (Ma et al., 2011). The resting membrane potential of iPSC-CMs have been reported as −68.1 mV (Van de Sande et al., 2021), compared to the *in vivo* human atrial and ventricular resting potentials of −70 mV and −87    mV, respectively (Trautwein et al., 1962). Comparing ventricular-like iPSC-CMs to human mature CMs, the action potential durations (APD) are similar, ranging from 100 to 400 ms depending on the type of CM (Luo and Rudy, 1994; Shih, 1994; Ma et al., 2011). Differences in the AP between iPSC-CMs and human mature CMs arise from changes to upstroke velocity, waveform (lack of phase 1 in iPSC-CMs), and a less pronounced depolarization plateau.

#### 2.1.1 *I*_Na_

The sodium channel Na_v_1.5 contributes to the same phase 0 (depolarization) of the AP in both iPSC-CMs and human CMs. The activation and inactivation kinetics and current densities are similar between iPSC-CM and *in vivo* cardiomyocytes (Ma et al., 2011). Interestingly, recovery from inactivation is faster in iPSC-CMs, with fast (τ_f_) and slow (τ_s_) recovery time constants of 2.58 ± 0.31 ms and 47.17 ± 7.01 ms (Selga et al., 2018), compared to atrial CMs isolated from the adult heart (τ_f_ = 7.5 ms, τ_s_ = 61.4 ms) (Sakakibara et al., 1992). Recovery time constants reported from human ventricular CMs equally showed slower values than iPSC-CMs (τ_f_ = 5 ms, τ_s_ = 67.2 ms) (Sakakibara et al., 1993). In contrast, the upstroke velocity of iPSC-CMs was reported to be 50% slower than in cells from human heart tissue, highlighting a critical inherent difference observed between iPSC-CM sources and differentiation/maturation methods (Zhang et al., 2009; Ma et al., 2011; Davis et al., 2012). Other reports show an even slower iPSC-CM upstroke velocity of ∼50 V/s (Lundy et al., 2013) compared to CMs found in the human adult ventricular free wall at ∼250 V/s (Drouin et al., 1995). These findings may partially explain the slower beat rate commonly observed in iPSC-CMs (although other factors are involved, like maturation state).

**FIGURE 1:**
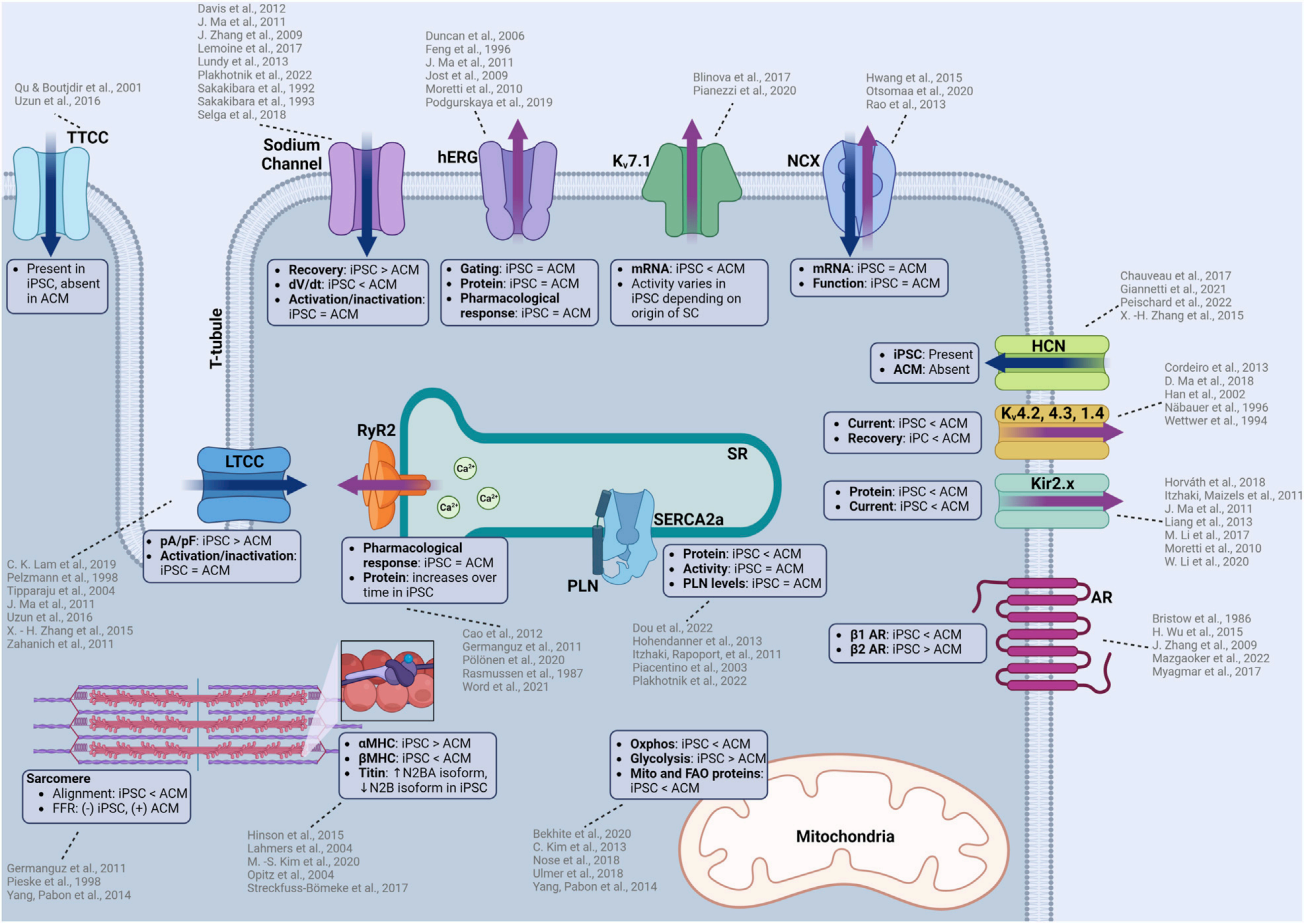
Summary of functional similarities and differences between iPSC-CMs and adult human CMs (ACM). iPSC-CMs are similar in many aspects including electrophysiology, calcium handling, contractile machinery which allows the use of iPSC-CMs to predict cellular responses to novel compounds. However, there are some differences that must be considered when considering iPSC-CMs as a model for cardiotoxicity testing. Some of these differences include ion channel kinetics, upstroke velocities, structural differences in the contractile machinery, isoform of proteins, and metabolic function. TTCC: T-type calcium channel, LTCC: L-type calcium channel, hERG: human-ether-a-go-go (rapid delayed rectifier potassium channel), Kv7.1: slow delayed rectifier postassium channel, NCX: sodium calcium exchanger, HCN: hyperpolarization-activated cyclic nucleotide gated channel, K_v_4.2, 4.3, 1.4: voltage dependent potassium channels responsible for the transient outward current, AR: adrenergic receptor, RyR2: ryanodine receptor, SERCA2a: sarcoendoplasmic reticulum calcium ATPase, PLN: phospholamban, Oxphos: oxidative phosphorylation, MHC: myosin heavy chain. Created with www.biorender.com.

Despite these differences, iPSC-CMs are very sensitive in detecting compound effects on N_av_1.5, and iPSC-CMs sodium channels have similar tetrodotoxin sensitivity compared to adult human CMs (Lemoine et al., 2017) (Figure 1). iPSC-CMs can recapitulate the electrophysiological characteristics of sodium channelopathies allowing for therapeutic testing using patient-derived cells (Davis et al., 2012) or incorporated into platforms that can accurately measure conduction velocity for compound testing (Dou et al., 2020). The robustness of the sodium channels in iPSC-CMs is further exemplified by the measurement of previously unknown cardiotoxic mechanisms of local anesthetics (Plakhotnik et al., 2022).

#### 2.1.2 *I*_Ca_

Most calcium currents in iPSC-CMs originate from the L-type calcium channel (Ca_v_1.2) but residual currents from T-type calcium channels exist related to the maturation process for the cells.

L-type calcium channels (LTCC) are present in iPSC-CMs and the cells have the expected response to nifedipine, a specific LTCC inhibitor, which causes cessation of spontaneous beating at 200 nM (Zahanich et al., 2011). Another independent study confirmed channel inhibition with a 20% reduction in spontaneous *I*_Ca_ through nifedipine inhibition (Zhang et al., 2015), emulating the pharmacological response of adult heart CMs. The iPSC-CM L-type calcium channels have similar activation and inactivation characteristics to adult human CMs (Ma et al., 2011; Uzun et al., 2016) and have similar or higher transcript or protein levels to the adult human heart (Ivashchenko et al., 2013; Rao et al., 2013; Fernández-Morales et al., 2019). While similarities exist, the *I*_Ca_ density is larger than human CMs (Ma et al., 2011), when compared to atrial CMs (Tipparaju et al., 2004) and CMs obtained from Tetralogy of Fallot hearts (Pelzmann et al., 1998). The patient-specific sensitivity of LTCC inhibition was modeled in iPSC-CMs, including observed reductions in the transcripts for contractility related genes (TNNI3, MYH7) after verapamil treatment (Lam et al., 2019). These findings demonstrate how iPSC-CMs can accurately detect patient-specific functional and transcriptomic effects as a result of calcium channel antagonists.

T-type calcium channels (Ca_v_3.1/2) are present in the embryonic stages of the heart but diminish over time and are absent in the adult heart (Qu and Boutjdir, 2001). Interestingly, iPSC-CMs are reported to have T-type calcium channel expression which contributes to *I*_Ca_ (Uzun et al., 2016). The presence of these channels is a sign of cell immaturity, and their presence should be considered when investigating modulators of the calcium flux as the observed effect could result from a mix of altered L- and T-type currents (Figure 1).

#### 2.1.3 *I*_Kr_ and *I*_Ks_

Rapid and slow delayed rectifier currents are generated by the K_v_11.1 (also known as the human ether-a-go-go channel, or hERG) and K_v_7.1 potassium channels, respectively. Both potassium channels are present in iPSC-CMs (Moretti et al., 2010), with similar gating properties compared to adult human CMs (Feng et al., 1996; Jost et al., 2009; Ma et al., 2011). Pharmacological testing revealed that *I*_Kr_ in iPSC-CMs are sensitive to erythromycin (Podgurskaya et al., 2019), a known hERG channel antagonist and arrhythmogen (Duncan et al., 2006). The sensitivity of iPSC-CMs toward hERG channel antagonists make them a valuable model for pharmacological proarrhythmic risk assessment as the hERG channel is the canonical target for arrhythmogenesis from QT-prolongation.

Differences in potassium currents between iPSC-CM and adult cardiomyocytes originate from an alteration in *I*_Ks_, where lower levels of K_v_7.1 (KCNQ1) mRNA in iPSC-CMs was measured compared to adult ventricular CMs (Blinova et al., 2017) (Figure 1). In addition to reduced expression, the activity of K_v_7.1 can vary depending on the origin of the stem cell (even when using different source tissue within the same patient). The highest activity of *I*_Ks_ was measured in iPSC-CMs derived from cardiac mesenchymal progenitor cells, followed by cells derived from bone marrow mesenchymal stem cells, with the lowest activity in cells derived from dermal fibroblasts (Pianezzi et al., 2020). The exact etiology for these differences in source tissue is not clear. In adult CMs, *I*_Ks_ is known to be modulated by adrenergic signaling where the activity is increased through PKA dependent phosphorylation (Terrenoire et al., 2005). This finding is illustrated by action potential duration increases in human ventricular tissue when exposed to the β-blocker sotalol (Jost et al., 2005). Similar adrenergic-dependent increases in *I*_Ks_ activity are observed in iPSC-CMs in response to sympathetic stimulation (Moretti et al., 2010; Zhang et al., 2014).

#### 2.1.4 *I*_K1_

The inward rectifying potassium current is primarily driven by the Kir2.x subfamily of potassium channels. Very low Kir2.x protein and *I*_K1_ current was detected in iPSC-CMs, compared to adult heart tissue (Ma et al., 2011; Li et al., 2020), illustrating that overexpression of Kir2.x channels (Li et al., 2017) may be necessary to increase *I*_K1_ current to better assess the risk of QT-prolongation (Figure 1). One report showed that the *I*_K1_ current was similar to human right atrial (RA) and left ventricular (LV) tissue (Horváth et al., 2018), and argued that the current differences others have reported can be attributed to the lower resting membrane potential of iPSC-CMs (Moretti et al., 2010; Itzhaki and Maizels, 2011; Liang et al., 2013). While possible, the low levels of Kir2.x protein and *I*_K1_ current can also be explained by the typical location of Kir2.x channels in T-tubules, a structure commonly absent in immature iPSC-CMs without maturation protocols (Clark et al., 2001).

#### 2.1.5 *I*_f_

The *I*_f_ is the pacemaker current generated from the hyperpolarization-activated cyclic-nucleotide gated channel (HCN) commonly found in the sinoatrial node, atrioventricular node, and Purkinje fibers of conductive tissue. The presence of *I*_f_ in iPSC-CMs is a result of its automaticity and a sign of heterogeneity as healthy adult human ventricular CMs do not have HCN channels or *I*_f_ (Peischard et al., 2022). The *I*_f_ in iPSC-CMs is persistent, even with longer cultures and maturation, demonstrating a typical mixed iPSC-CM population (ventricular, atrial, and nodal) (Giannetti et al., 2021), although the spontaneous rate does decrease as culture time increases. As specialized differentiation protocols become more widely available, ventricular-only iPSC-CM populations (or atrial or nodal) allow for HCN expression in the proper end-differentiated cell desired (Figure 1). There are some disagreements regarding the function of the HCN channel. Initially Zhang et al. reported that ivabradine, a specific HCN channel inhibitor, had no effect on the automaticity in iPSC-CMs (Zhang et al., 2015). More recently, Chauveau et al. demonstrated that ivabradine had a concentration-dependent effect on slowing the automaticity of iPSC-CMs until complete cessation (Chauveau et al., 2017). The differences in their observations could be a result of altered differentiation or maturation protocols, illustrating that the *I*_f_ in iPSC-CMs needs to be carefully characterized for relevant studies.

#### 2.1.6 *I*_to_

The *I*_to_ is driven by K_v_4.2, K_v_4.3, and K_v_1.4 potassium channels, which contribute to the phase 1 “notch” in the cardiac AP. When measuring *I*_to_ in patch clamping experiments with iPSC-CMs, phase 1 of the AP is often subtle or missing, but was exacerbated through increased pacing (Cordeiro et al., 2013). Recovery from activation of the potassium channels responsible for *I*_to_ are generally slower (Cordeiro et al., 2013) when compared to human epicardial ventricular CMs (Wettwer et al., 1994) and the current densities of *I*_to_ were observed to be ∼50% of those found in human sub-epicardial ventricular CMs (Näbauer et al., 1996; Ma et al., 2018) (Figure 1). Interestingly, there were higher currents observed in 20% of the cells, which points to the heterogenous population of cells in mixed differentiated iPSC-CM cultures. Temperature during patch clamp experiments may additionally be a factor as K_v_4.2, K_v_4.3, and K_v_1.4 potassium channel activity can be temperature sensitive (Han et al., 2002). These channel activity differences demonstrate some of the reasons why the APs are partially inconsistent between iPSC-CMs and adult CMs.

### 2.2 Calcium cycling

#### 2.2.1 T-tubules

iPSC-CMs have high amounts of calcium flux, indicative of the presence of calcium-induced calcium release (CICR) to facilitate contraction. An important structural component of CICR are the T-tubules, invaginations into CMs for spatial positioning of ion channels for efficient ion conduction. Unmatured iPSC-CMs are known to lack T-tubules, a detriment to efficient CICR (Lieu et al., 2009; Gherghiceanu et al., 2011; Yang et al., 2014). The absence of T-tubules are confirmed through electron microscopy (Gherghiceanu et al., 2011; Lundy et al., 2013). As T-tubules develop over time in the human heart throughout fetal development (Ziman et al., 2010), the general immaturity of iPSC would explain the absence of T-tubules. However, there are many ways to enhance T-tubule formation in iPSC-CMs through maturation protocols, thereby positively contributing to the calcium handling capabilities. The effects of t-tubule formation as iPSC-CMs undergo maturation protocols will be explored in a later section.

#### 2.2.2 Junctophilin-2

Junctophilin-2 (JP2) is a structural protein that tethers the T-tubule to the sarcoplasmic reticulum (SR) to spatially orient LTCC and ryanodine receptors (RyR) for coordinated CICR (Takeshima et al., 2000). iPSC-CMs express fully functional JP2 and there are increases in expression level proportional to culture time as a function of maturation (Pretorius et al., 2021; Weninger et al., 2022). JP2 found in iPSC-CMs is cleavable by calpain (Weninger et al., 2022), which is a known target of proteolysis in human hearts during cardiac injury (Murphy et al., 2013; Guo et al., 2015; Chan et al., 2019). Intrinsic changes in JP2 function in iPSC-CMs has not been explored significantly, but one study did generate patient-specific iPSC-CMs with a JP2 T161K variant and demonstrated an arrhythmogenic phenotype (Valtonen et al., 2023). The functional expression of JP2 in iPSC-CMs and the ability to recapitulate genetic variant phenotype could prove iPSC-CMs as a valuable model for developing therapeutics targeting JP2.

#### 2.2.3 Ryanodine receptors

RyRs facilitate calcium release from the SR upon calcium binding. iPSC-CMs express functional RyR (Germanguz et al., 2011) with its expression increasing as the cells are matured (Cao et al., 2012). The RyR expressed in iPSC-CMs is able to emulate RyR mutations and shows responsiveness to modulators such as carvedilol, which inhibit the phosphorylation of RyR though β-adrenergic inhibition (Pölönen et al., 2020); demonstrating an ability to detect physiologically relevant effects on CICR (Figure 1). While RyR function in iPSC-CMs appears to be identical to Ryr in adult human CMs, iPSC-CM RyR response to caffeine is much lower (Rasmussen et al., 1987; Germanguz et al., 2011), indicative of potential binding kinetic and activity differences. This lower response should be considered if total SR calcium release is to be measured using caffeine stimulation. The efficacy of a novel RyR2 inhibitor, EL20, for the treatment of catecholaminergic polymorphic ventricular tachycardia was assessed using patient-derived iPSC-CMs (Word et al., 2021). The drug was found to have an IC_50_ of 82 nM and it successfully suppressed arrhythmogenic activity while maintaining homeostatic calcium handling within the iPSC-CM, illustrating a potential for precision therapeutic testing.

#### 2.2.4 Sarco-endoplasmic reticulum calcium ATPase (SERCA-2a) and phospholamban (PLN)

SERCA-2a is the primary channel for the reuptake of calcium into the SR after contraction and is lower in both activity and expression level in heart failure (Figure 1). SERCA-2a activity can be negatively modulated by PLN through allosteric binding. The presence of SERCA-2a was verified by measuring calcium fluxes within iPSC-CMs with activity inhibited by thapsigargin, a channel-specific antagonist (Itzhaki and Rapoport, 2011). Overall, calcium handling characteristics are similar to those found in the human heart and have been extensively characterized (Itzhaki and Rapoport, 2011; Dou et al., 2022). Some minor differences include lower levels of SERCA-2a expression but similar levels of PLN transcripts compared to the adult human heart (Rao et al., 2013). SERCA-2a activity in iPSC-CMs has been quantified, with a time decay constant tau reported as ∼0.1 s (Plakhotnik et al., 2022), comparable to those measured in rabbit heart ∼0.07 s (Wang et al., 2021) but faster than rat hearts (0.3 s) (Mohamed et al., 2013). A similar tau value of 0.209 s was reported in human non-failing ventricular CMs (Piacentino et al., 2003). Interestingly, the tau in healthy human ventricular CMs was reported as 0.51 s (Hohendanner et al., 2013), which indicates lower SERCA-2a activity than iPSC-CMs. However, this phenomenon is likely be due to regional differences in cytosolic calcium release as discussed in the study manuscript.

#### 2.2.5 Sodium calcium exchanger (NCX)

Transcript levels of NCX in iPSC-CMs are comparable to those found in the adult human heart (Rao et al., 2013). NCX antiporters in iPSC-CMs have robust function and even contribute to calcium removal during diastole (Hwang et al., 2015). The utility of iPSC-CMs was demonstrated when an inhibitor of NCX, ORM-11372, was discovered and characterized (Otsomaa et al., 2020). The IC_50_ for the outward current in iPSC-CMs was 4.8 nM, compared to an IC_50_ of 11.3 nM in rat primary ventricular CMs, a nearly three-fold change to be considered when observing intracellular calcium or sodium homeostasis.

### 2.3 Cellular signaling

#### 2.3.1 Calmodulin

Calmodulin is a calcium-responsive modulator of downstream kinases, importantly involved in responses to calcium signaling. Calmodulin signaling is present in iPSC-CMs and can be manipulated by changing calcium levels to emulate human disease phenotypes (Rocchetti et al., 2017; Liu et al., 2021). The similarity of calmodulin signaling between iPSC-CMs and adult human CMs can be utilized to investigate calmodulin modulators as a therapy for heart failure, and assessing any potential cardiotoxic effects associated with alterations in protein activity (Schulman and Anderson, 2010).

#### 2.3.2 Adrenergic signaling

Adrenergic signaling in iPSC-CM is similar to adult human CMs. Isoproterenol, a canonical β-adrenergic agonist, induces a predictable positive chronotropic (two-fold increase in beating rate at 1 μM drug concentration) and inotropic (contractility) response (Zhang et al., 2009; Wang et al., 2018; Dou et al., 2023). However, a major difference between iPSC-CMs and *in vivo* CM response is that the positive chronotropic and inotropic effect of isoproterenol is primarily modulated through β2 adrenergic receptors in unmatured iPSC-CMs (Wu et al., 2015), compared to β1 receptors in the human heart (Bristow et al., 1986; Myagmar et al., 2017). The difference in adrenergic receptor subtype could contribute to changes in sensitivity to any cardiotoxic compounds that affect the adrenergic system. Downstream effectors of adrenergic signaling, such as the cAMP/PKA pathway, are present in iPSC-CMs and play an important role in modulating calcium release and automaticity (Mazgaoker et al., 2022).

### 2.4 Contractile machinery

The ability of iPSC-CMs to contract make it possible to quantify compound-induced changes to inotropy (Wang et al., 2020). There are several differences in the contractile machinery between iPSC-CMs and the human heart. Even with maturation, the sarcomere organization of iPSC-CMs is more disorganized, with misaligned actin, and the cells presenting a more spherical (compared to rectangular) form (Yang et al., 2014). However, despite the differences in cellular morphology and actin structure, the average sarcomere length of ∼1.98 μm in iPSC-CMs (Lee et al., 2012) were similar to the measurements of samples acquired from human ventricular biopsies (Herron et al., 2006). A fundamental property of the human heart is the force-frequency relationship, an endogenous mechanism of the heart to match the frequency of beating to the contractile force (Endoh, 2004). iPSC-CMs exhibit a negative force-frequency relationship which is more akin to the level of contractility in heart failure (Pieske et al., 1998; Germanguz et al., 2011), but possess mild post-rest potentiation, a phenomenon where contraction after rest becomes stronger in adult CMs (Germanguz et al., 2011). Contractile forces of iPSC-CMs in 3D constructs were lower (∼0.08 mN/mm^2^) (Tulloch et al., 2011) compared to human myocardial strips (44 ± 11.7 mN/mm^2^) (Hasenfuss et al., 1991). Single cell contractile forces of iPSC-CMs were reported to be in the range of 0.1nN–144 nN depending on experimental conditions (Rodriguez et al., 2014), which is lower than measurements made in adult rat CMs (Yasuda et al., 2001; Nishimura et al., 2004) and human ventricular CMs (Van Der Velden et al., 1998).

Cardiac troponin, β-myosin heavy chain, and myosin light chain protein levels are similar to or slightly lower than the levels in the human fetal heart (Rao et al., 2013). There is higher α-myosin heavy chain levels in iPSC-CMs compared to adult CMs, which is converted to β-myosin heavy chain as the iPSC-CMs mature (Kim et al., 2020), thereby emphasizing the importance of maturation protocols to ensure the function of the iPSC-CMs can more closely recapitulate adult CM function. Titin isoforms are also different in iPSC-CMs, as human hearts have a shift from the more compliant larger N2BA isoform to the smaller and stiffer N2B isoform post-natally (Lahmers et al., 2004; Opitz et al., 2004). While iPSC-CMs express titin, it is predominantly in the N2BA isoform (Hinson et al., 2015; Streckfuss-Bömeke et al., 2017). The isoform differences do not negatively affect the ability to create iPSC-CM models that can recapitulate phenotypes associated with pathological titin changes (Hinson et al., 2015), demonstrating that small molecule modulation of titin would likely be detected. Similar to isoform differences in β-myosin heavy chain, adult cardiac troponin I (cTnI) levels in iPSC-CMs were found to be absent or lower in favor of slow skeletal troponin I (ssTnI) (Miki et al., 2021) which is an isoform found in the developing hearts that eventually switches to adult cTnI as the heart matures (Saggin et al., 1989; Hunkeler et al., 1991).

### 2.5 Metabolism

In the adult human heart, 95% of the ATP is generated through oxidative phosphorylation utilizing fatty acids as a primary carbon source (Lopaschuk et al., 2021). In iPSC-CMs, the baseline metabolic profile is primarily glycolytic, but fatty acid can be utilized for ATP generation, especially with cell maturation and forced utilization of mitochondrial metabolism (Kim et al., 2013; Yang et al., 2014). Quantification of mitochondrial and fatty acid oxidation-related proteins and transcripts reveals lower amounts in iPSC-CM (before maturation) compared to the adult human heart (Nose et al., 2018; Ulmer et al., 2018; Bekhite et al., 2020). Furthermore, iPSC-CMs express higher levels of fetal glucose transporters (GLUT1) opposed to the adult form (GLUT4) (Bowman et al., 2019). After maturation protocols, expression of mitochondrial proteins, oxygen consumption, and fatty acid utilization are all increased, indicative of a successful shift and maturation of the metabolic phenotype towards a more adult-like state (Nose et al., 2018; Ulmer et al., 2018; Bekhite et al., 2020).

## 3 Considerations when using iPSC-CMs for cardiotoxicity testing

### 3.1 Maturation

**FIGURE 2:**
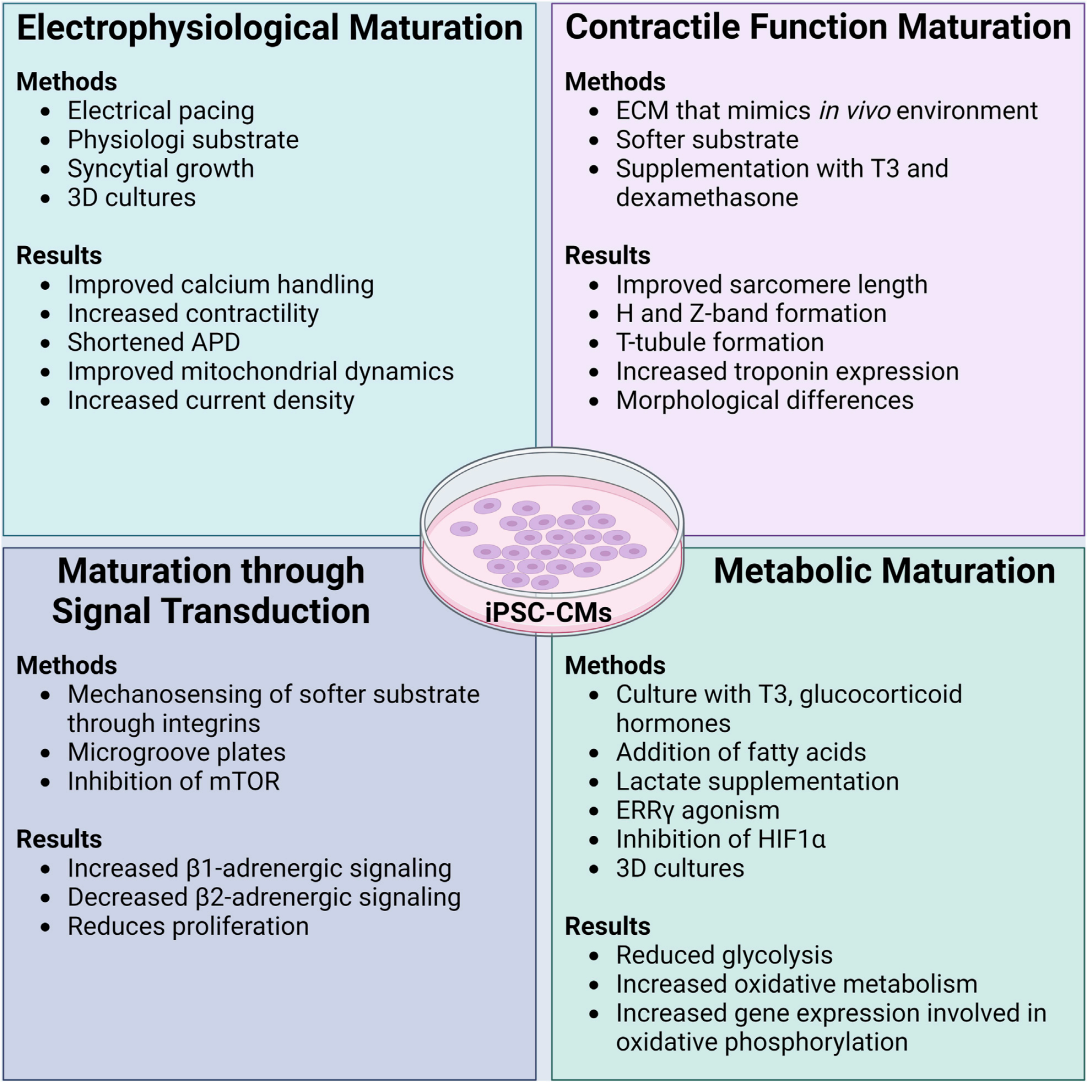
Methods and functional results of maturation protocols in iPSC-CMs. Initially differentiated iPSC-CMs are known to have an immature phenotype akin to a neonatal CM. Many different maturation protocols have been developed to ensure that functional parameters and the cellular phenotype of iPSC-CMs can more closely and accurately reflect that of an adult human CM. In general, maturation protocols should aim to improve the electrophysiology, contractility, signal transduction, and metabolism of iPSC-CMs. While the combination of different maturation protocols leads to the best results of functional improvement in iPSC-CMs, it appears that culturing iPSC-CMs on soft substrates with extracellular matrix coating that is similar to the *in vivo* environment is imperative. ADP: action potential duration, ECM: extracellular matrix, T3: thyroid hormone T3, mTOR: mammalian target of rapamycin, ERRγ: estrogen related receptor γ, HIF1α: hypoxia inducible factor-1α. Created with www.biorender.com.

As described, the phenotype exhibited by iPSC-CMs after initial differentiation is closer to a human fetal/neonatal cell, not fully reflective of the physiological characteristics of an adult CM (Van Den Berg et al., 2015). It is important to consider how maturation can improve these aspects of cellular physiology in order to acquire a better representation of potential cardiotoxic effects (Figure 2).

#### 3.1.1 Electrophysiological maturation

A mature electrophysiological system increases reliability when assessing a candidate compound for arrhythmogenic effects. Several methods improve the electrophysiological function of iPSC-CMs. Currently, the most common method of electrophysiological maturation is by pacing iPSC-CMs with an electrode, improving calcium handling and contractile function (Wu et al., 2021; Shen et al., 2022).

Improvements to (increasing) the AP upstroke velocity in iPSC-CMs was made through maturation by plating the cells on substrates possessing a stiffness more akin to a healthy heart (i.e., softer than normal tissue culture plastic), with a scaffold/extracellular matrix (ECM) composed of polydimethylsiloxane (PDMS) and Matrigel (Herron et al., 2016). In addition to ECM composition, cell density plays a role in the maturity of electrophysiology. Syncytial growth (multinucleate cell formation) of iPSC-CMs with proper cell-cell contact can improve the *I*_K1_ current and the expression Kir2.1 channels (Li et al., 2020). Some groups have created three-dimensional constructs for denser cell-cell connections, improving upstroke velocity through increases in *I*_Na_ density, resulting in an AP waveform that more closely resembles those observed in LV tissue (Lemoine et al., 2017). Other 3D microtissue techniques saw improved electrical coupling between the cells and the formation of refined intercalated disks (Giacomelli et al., 2020). Interestingly, overexpression of the *K*_ir_2.1 channel improved functional parameters, such as a shortened APD, an increased upstroke velocity, and improvements to calcium signaling and mitochondrial dynamics (Zhou et al., 2023). While this approach is novel and shows signs of promising improvements in electrophysiology, the overexpression could negatively alter the homeostatic balance of (intracellular) ions with unknown consequences (Nerbonne et al., 2001). The endoplasmic reticulum unfolded protein response, mediated through protein kinase-like ER kinase (PERK) and inositol-requiring protein-1 (IRE1), can have negative effects on Na_v_1.5 and hERG expression in CMs (Liu et al., 2018). The inhibition of these pathways could evoke electrophysiological maturation.

Other novel approaches to electrophysiological maturation include pharmacological additions of SGLT2 inhibitors, which resulted in an increase in *I*_Na_ and *I*_K1_ (Dago et al., 2022). Co-culturing iPSC-CMs with Kir2.1 expressing human embryonic kidney (HEK) cells to create an electrical syncytium where the HEK cells acted as a pacer, resulted in decreased spontaneous beating (of the cardiomyocytes) and an increase in contraction amplitude (Costa et al., 2021). Whether this technique confers an advantage over more common maturation methods of pacing is unclear.

#### 3.1.2 Maturation through signal transduction

The importance of signal transduction can be attributed to mechanosensing through integrin signaling. iPSC-CMs plated on PDMS and Matrigel ECM showed improvements in conduction velocity (55 cm/s), similar to adult LV CMs (Herron et al., 2016).

As discussed, iPSC-CM adrenergic function is partially mediated through β2-adrenergic receptors, as opposed to relying solely on β1-adrenergic receptors like in the adult heart. This change can be addressed as the increasing β1-adrenergic signaling and decreasing β2-adrenergic signaling are a function of maturation (Jung et al., 2016). The shift in adrenergic signaling can be achieved through biomechanical stimulation, such as culturing iPSC-CMs on PDMS micro-grooved devices (Jung et al., 2016).

Another signaling pathway that modulate the maturity of iPSC-CMs is the mTOR pathway. Inhibition of mTOR leads to improved contractility and more adult-like metabolic and electrophysiological profiles (Garbern et al., 2020). The exact mechanism for this effect is still unknown but the authors speculate that it can be attributed to the inhibition of proliferative signals and switching the iPSC-CMs to a quiescent state to mimic the low proliferative potential of adult CMs.

#### 3.1.3 Maturation of contractile function

Maturation of iPSC-CMs leads to the development and refinement of the contractile machinery through increased sarcomere length, with distinct H and Z-bands (Lundy et al., 2013). A method commonly used to mature contractile function is to plate iPSC-CMs on a substrate with a Young’s modulus more similar to the *in vivo* environment (like PDMS), coated with ECM proteins (i.e., gelatin, collagen, fibronectin) (Wang et al., 2018; Knight et al., 2021; Körner et al., 2021; Dhahri et al., 2022). The addition of a softer substrate results in rod-shaped cells and more organized actin filaments (Lemoine et al., 2017). Furthermore, addition of microgrooves within the substrate can enhance sarcomere alignment and improve contractility (Dou et al., 2021; 2023). Increases in mechanical outputs (as measured by sarcomere shortening) were found by utilizing a softer substrate for culture and cell maturation (Ribeiro et al., 2015).

Improvements to the CICR can be made through the maturation of T-tubules and calcium handling proteins. Supplementation with thyroid hormone T3 (100 nM) and dexamethasone (1 mM) on iPSC-CMs plated on a Matrigel coating (3 mL media/well) showed enhanced formation of T-tubules, which are essential for efficient CICR (Parikh et al., 2017). The formation of T-tubules was attributed to the function of bridging integrator-1 (BIN1), which contributes to the co-proximity of LTCC and the RyR (Guo et al., 2022). This raises the possibility of modulating BIN1 activity for further CICR maturation. T-tubule formation can be enhanced through supplementation with an estrogen-related receptor gamma (ERRγ) agonist (e.g., T112 at 3 μM), in conjunction with an S-phase kinase-associated protein inhibitor (e.g., T623 at 40 nM) (Miki et al., 2021). This approach has the added benefit of increasing the expression of adult troponin I (TNNI3) but did not appear to reduce fetal troponin I (TNNI1). Within this study, the iPSC-CMs were still spherical in morphology, a sign of continued immaturity and indicating that the agonist/antagonist alone is not enough for full maturation of the contractile machinery. As maturation progresses, increases in SERCA-2a activity and calcium cycling are observed, although a plateau is reached after 21 days of culture (Hwang et al., 2015).

A softer substrate and ECM are beneficial for maturation to occur, and modulation of specific signaling pathways is beneficial to the maturation of the contractile function in iPSC-CMs. When comparing cells grown on glass (25 GPa) and PDMS (1.5 kPa, 15kPa, 28 kPa) surfaces, iPSC-CMs grown on PDMS had improved contractility and calcium handling, similar to those of an adult CM (Körner et al., 2021). Similarly, culturing iPSC-CMs on softer substrates have yielded improvements in conduction velocity (doubling to 43.6 ± 7 cm/s), structural maturation (gap junction formation and hypertrophic growth), and activation of integrin signaling pathways (Herron et al., 2016). While the signs of maturation as a result of a softer substrate are substantial, the optimal combination of maturation protocols remains to be elucidated and standardized.

Culturing iPSC-CMs in 3D scaffolds is another approach used to mature contractile function through the organization of myofibrils and better spatial positioning of CICR related proteins (Silbernagel et al., 2020; Pinton et al., 2023). These approaches have demonstrated success in the formation of 3D structures more akin to that of heart tissue.

#### 3.1.4 Metabolic maturation

Metabolic maturation of iPSC-CMs is often overlooked and can take up to 200 days for maximal effect (Ebert et al., 2019). Many protocols suggest culturing iPSC-CMs with thyroid and glucocorticoid hormones (Yang et al., 2014; Birket et al., 2015; Parikh et al., 2017) and fatty acids (Rana et al., 2012; Horikoshi et al., 2019; M. Feyen et al., 2020) thereby reducing the glycolytic phenotype and promoting fatty acid uptake/utilization. Lactate supplementation additionally shows an improvement in the metabolic profile, along with an increased expression of genes involved in oxidative phosphorylation (PPARα, CPT-1B, PGC-1α, PDK4) (Bekhite et al., 2020).

In addition to the positive effects ERRγ agonism can have on iPSC-CM contractility, ERRγ is a regulator for the metabolic shift towards oxidative metabolism (Alaynick et al., 2007). Adding an ERRγ agonist (T112 at 3 μM) increased oxygen consumption in iPSC-CMs, indicative of enhanced mitochondrial function and metabolic maturation (Miki et al., 2021). Additionally, inhibition of hypoxia induced factor-1α and lactate dehydrogenase A can cause partial shifts of iPSC-CM metabolism from aerobic glycolysis to fatty acid oxidation (Hu et al., 2018).

Considering structural approaches, three dimensional engineered heart tissue showed robust maturation of metabolism through increases in ATP synthesis and an upregulation of fatty acid oxidation-related proteins (Ulmer et al., 2018). 3D iPSC-CM engineering approaches with iPSC-CMs have gained traction and show promising signs of maturation in many aspects of cellular physiology. The advantages and applications of 3D iPSC-CM cultures have been discussed in other reviews (Zuppinger, 2019; Cho et al., 2021; Liu et al., 2022).

### 3.2 Sources of iPSC-CMs

iPSC-CMs can be derived from different tissues, resulting in altered responses to tested compounds (even when from the same individual). Commercial sources offer cells that are quality controlled, with pre-defined plating efficiencies, and are delivered already differentiated into CMs with high purity. While commercial sources offer several advantages, there is some evidence showing batch-to-batch variability (Nozaki et al., 2016; Mannhardt et al., 2017). A comparison of the effects of pro-arrhythmic drugs from two different lots of commercial iPSC-CMs showed terfenadine (potent *I*_*Kr*_ inhibitor) induced early after depolarizations (EADs) in one but not the other cell source (Nozaki et al., 2016). Other drugs tested in this study showed no variability between the different cell lots. Despite the variance in the production of EADs, terfenadine still produced similar changes to beat rate and FPDc, and the study concluded that lot-to-lot variability was within acceptable margins.

Other assessments of iPSC-CMs from 10 different sources (5 commercial, 5 academic) showed major differences in baseline metrics such as beat rate, contractile force, time to peak contraction, and relaxation time (Mannhardt et al., 2020). Despite these differences at baseline, the sarcomere lengths between all sources were similar and tested pharmaceuticals elicited similar responses, as measured by changes to contractile force. Although some cellular physiological differences will exist in cells from different sources, relative changes from baseline and data reproducibility are more important for drug evaluation.

Another source of iPSC-CMs are those derived from a specific patient with a desired phenotype/disease state. The phenotype of the resulting cells can be highly patient tissue specific, a detriment to the generalization of cardiotoxicity predictions. For example, atrial and ventricular fibroblast-derived iPSC-CMs were noted to have different field potential durations and higher action potential duration/conduction velocities (Wang et al., 2023) than iPSC-CM derived from dermal fibroblasts (Lan et al., 2020) or blood (Lam et al., 2020). The tissue sources of iPSC-CMs can also affect how fast and how well the resultant iPSC-CM can mature, as illustrated with iPSC-CMs derived from cardiac sources having more ion channels and calcium handling proteins than those derived from non-cardiac sources with the same duration of maturation (Pianezzi et al., 2020).

### 3.3 The heterogenous population of iPSC-CMs

Unless differentiation protocols are designed to produce a specific subset of CMs, the resultant population of iPSC-CMs can include a heterogenous mixture of nodal, atrial, ventricular, and Purkinje related cell types (Zhang et al., 2009; Ma et al., 2013). Although the majority of iPSC-CMs tend towards more ventricular-like phenotypes, the composition is important to determine what anatomical or cellular part of the heart a novel compound may be targeting. Maturation protocols can be customized to what type of iPSC-CMs is desired. For instance, a PPARα agonist, dexamethasone, T3, and palmitate in low-glucose media worked well for maturing iPSC-CMs towards a ventricular phenotype (cTNT+, MYL2+) (Funakoshi et al., 2021). For more atrial-like iPSC-CMs, retinoic acid was found to be important, along with lower BMP4 and activin-A concentrations (Goldfracht et al., 2020). Atrial-like iPSC-CMs were delineated with the absence of MLC2v and confirmed with electrophysiological measurements. Modulation of ion channels can be used to push iPSC-CM populations towards a pacemaker-like phenotype (Kleger et al., 2010).

Contractile force and frequency of beating are known to be different in atrial or ventricular iPSC-CMs (Zhao et al., 2019). The advantage of delineating the different populations was demonstrated where a small segment of engineered heart tissue on a “biowire” platform was created with both atrial and ventricular iPSC-CMs, effectively making a “mini heart”.

There are many variations on maturation protocols, and it is difficult to determine which one is objectively the most beneficial. It is evident that often maturing one aspect of iPSC-CM cellular function improves others concurrently, and no one technique can mature all aspects at once. Nonetheless, if functional metrics in electrophysiology, cell signaling, contractile function, calcium handling, and metabolism show maturation, it would support a reliable platform for reproducible and translatable results in cardiotoxicity testing.

## 4 Examples of drug testing using iPSC-CMs

The utilization of iPSC-CMs for cardiotoxicity testing is a growing area and is one of the main motives for the Comprehensive *In vitro* Proarrhythmia Assessment assay (CiPA) initiative, established by the Cardiac Safety Research Consortium, the Food and Drug Administration, and the Environmental Sciences Institute (Sager et al., 2014). CiPA was formed to address novel approaches in assessing drug-induced proarrhythmia risk with three major components: 1) assessing the effect of candidate drugs on crucial ventricular ion channel currents, 2) evaluation of potential effects on ion channels *in silico* for assessment of the cumulative effects on the cardiac AP, and 3) observing and quantifying discrepancies in a systems biology approach through the use of iPSC-CMs and human electrocardiograms (Colatsky et al., 2016).

The initiative has resulted in the advancement of consensus protocols for the use of iPSC-CM in large multisite studies for proarrhythmic analysis with high reproducibility and accurate predictions. A large study enrolled 10 laboratories around the world using two commercial sources of iPSC-CMs and were able to assess the arrhythmic risks in 28 blinded drugs at an 87% predictivity (Blinova et al., 2018). A similar undertaking was performed by the same group in 2017 using two different commercial iPSC-CM lines which demonstrated that iPSC-CMs are sensitive to hERG and LTCC channel blockers, but had variable responses to late *I*_*Na*_ blockers (Blinova et al., 2017). Another multi-site study included four iPSC-CM cell lines and 18 individual investigation sites, demonstrating the sensitivity of iPSC-CMs towards multi-channel blocking compounds for accurate predictions of proarrhythmic risk (Millard et al., 2018). The testing found that there were consistent concentration-dependent effects for all of the 8 drugs tested, including prolongation of beat period and field potential duration with the *I*_*Kr*_ blocker E-4031, or the shortening of BP and FPD with nifedipine. The authors did note that 8/18 (44%) of the independent studies failed to detect delayed repolarization with E-4031 and attributed it to inadequate drug exposure, poor culturing techniques, and differences in ion channel densities. These studies illustrate that iPSC-CMs have a great potential for assessing the proarrhythmic risk of compounds and are in line with the goals of the CiPA initiative.

There have not been large scale studies assessing other (non-arrhythmogenic) metrics of cardiotoxicity, but smaller studies have been performed. iPSC-CMs were used to assess the negative effects of the breast cancer drug trastuzumab on CM structure and function (Kitani et al., 2019). Patient-derived iPSC-CMs created from individuals with identified trastuzumab-induced cardiotoxicity were able to recapitulate patient-specific responses, demonstrating the utility of iPSC-CMs in precision medicine-type cardiotoxicity testing. The analysis of iPSC-CM responses to trastuzumab elucidated putative metabolic mechanisms that contribute to trastuzumab cardiotoxicity. In a separate study, a library of iPSC-CMs was generated from patients with various genetic and hereditary disorders. The extent of drug cardiotoxicity effects were quantified utilizing the derived cells, which accurately recapitulated the clinical susceptibilities observed (Liang et al., 2013).

## 5 Conclusion

iPSC-CMs are powerful tools for assessing the cardiotoxicity of novel or existing compounds due to their similarity in cellular physiology to endogenous CMs. They can be used to predict the effects of candidate compounds in discovery pipelines on the complex electrophysiology, cellular signaling, metabolism, and contractile machinery in the adult CM. However, it is crucial to acknowledge that when considering iPSC-CMs as a model for cardiotoxicity testing, there remains certain disparities that should be considered. The distinctions encompass variances in ion channel kinetics and function, structural differences in the contractile machinery, isoforms of proteins, and metabolic function. These considerations are especially important if the target of the drug is one that is known to be different between adult CM and iPSC-CMs as highlighted in this review. The integration of iPSC-CMs into preclinical testing pipelines holds potential to expedite the discovery and assess the safety of novel therapeutic compounds, resulting in greater bench to bedside efficiency. For wider implementation in pre-clinical drug discovery, the use of iPSC-CMs should be (minimally internally) standardized regarding culture conditions, maturation protocols, and the assessment of cellular subtype, to ensure reproducibility. The incorporation of iPSC-CMs represents a crucial step towards addressing challenges of compound attrition and improving the landscape of drug development.
